# Innate Immunity Evasion by Enteroviruses: Insights into Virus-Host Interaction

**DOI:** 10.3390/v8010022

**Published:** 2016-01-15

**Authors:** Xiaobo Lei, Xia Xiao, Jianwei Wang

**Affiliations:** 1 MOH Key Laboratory of Systems Biology of Pathogens, Institute of Pathogen Biology (IPB), Chinese Academy of Medical Sciences (CAMS) and Peking Union Medical College, Beijing 100730, China; fyleixb@126.com (X.L.); xiaoxia850801@126.com (X.X.); 2 Collaborative Innovation Center for Diagnosis and Treatment of Infectious Diseases, Hangzhou 310003, China

**Keywords:** enteroviruses, innate immune responses, pattern-recognition receptors, antiviral response evasion

## Abstract

*Enterovirus* genus includes multiple important human pathogens, such as poliovirus, coxsackievirus, enterovirus (EV) A71, EV-D68 and rhinovirus. Infection with EVs can cause numerous clinical conditions including poliomyelitis, meningitis and encephalitis, hand-foot-and-mouth disease, acute flaccid paralysis, diarrhea, myocarditis and respiratory illness. EVs, which are positive-sense single-stranded RNA viruses, trigger activation of the host antiviral innate immune responses through pathogen recognition receptors such as retinoic acid-inducible gene (RIG-I)-likeand Toll-like receptors. In turn, EVs have developed sophisticated strategies to evade host antiviral responses. In this review, we discuss the interplay between the host innate immune responses and EV infection, with a primary focus on host immune detection and protection against EV infection and viral strategies to evade these antiviral immune responses.

## 1. Enterovirus

Enteroviruses (EVs) belong to the *Enterovirus* genus of the family *Picornaviridae* and have been classified into twelve species according to the International Committee on Taxonomy of Viruses (ICTV). Although five species only infect animals (EVE–J), the remaining seven species are known to infect humans, *i.e.* EV A–D and rhinovirus A–C (RV A–C). These species include hundreds of serotypes, such as polioviruses (PV), coxsackieviruses A and B (CV-A and -B), echoviruses, numbered EVs, and human rhinovirus (HRVs), among them including several important human pathogens such as PV, CV-A16, CV-B3, EV-A71, EV-D68, and HRV (Table 1) [1].

### 1.1. Pathogenesis

Human EV infections are widespread. EVs are the most common cause of aseptic meningitis, pericarditis, myocarditis and respiratory infections [2]. Generally, EV infections are asymptomatic (about 50%–80%) or cause clinically mild and self-limited infections [2]. However, some species can cause severe and potentially fatal infections. For instance, PV can invade the nervous system and cause poliomyelitis in children, the most significant disease caused by an enterovirus. CV can cause acute clinical manifestation ranging from mild febrile illness to more severe conditions including meningo-encephalitis, pancreatitis, and fulminate sepsis in neonates [3]. Some chronic diseases are also caused by CV, such as chronic myocarditis and type I diabetes [4,5].

**Table 1:** Classification of human enteroviruses [1].

Enterovirus Species	Type
*Human enterovirus A*	Human coxsackievirus A2–8, 10, 12, 14, 16.
	Human enterovirus 71, 76, 89–92, 114, 119–121.
*Human enterovirus B*	Human coxsackievirus A-9, B1-6.
	Human echovirus 1–9, 11–21, 24–27, 29–33.
	Human enterovirus 69, 73–75, 77–88, 93, 97–101, 106, 107, 110–113.
*Human enterovirus C*	Human coxsackievirus A1, 11, 13, 17, 19–22, 24.
	Human poliovirus 1–3.
	Human enterovirus 95, 96, 99, 102, 104, 105, 109, 116–118.
*Human enterovirus D*	Human enterovirus 68, 70, 94, 111, 120.
*Rhinovirus A*	Human rhinovirus A1, 2, 7–13, 15, 16, 18–25, 28–34, 36, 38–41, 43, 45–47, 49–51, 53–68, 71, 73–78, 80–82, 85, 88–90, 94, 96, 100–109.
*Rhinovirus B*	Human rhinovirus B3-6, 14, 17, 26, 27, 35, 37, 42, 48, 52, 69, 70, 72, 79, 83, 84, 86, 91–93, 97, 99, 100–106.
*Rhinovirus C*	Human rhinovirus C1-55.

Treatment or prevention of EV infections is challenged by the diversity of species that cause the same illness. For example, HRV infection can result in respiratory diseases including the common cold as well as more serious lower respiratory infections that exacerbate asthma [6,7]. However, there are three species with more than 100 HRV serotypes that cause these infections. Thus, developing a single vaccine against so many serotypes is nearly impossible [8]. Furthermore, similar respiratory illnesses including mild upper respiratory infections, pneumonia, and acute flaccid myelitis can also be caused by EV-D68 [9,10,11], which was identified in California, USA in 1962 [12]. Although EV-D68 has been rarely reported in the past 40 years, it has recently caused a epidemic in 2014 in the United States [10], leading to more than 1000 cases of severe respiratory disease.

Similarly, at least 23 EV serotypes can cause hand, foot, and mouth disease (HFMD), a common infectious disease of infants and children [13,14,15]. While EV-A71 and CV-A16 are the main causative agents of this disease, these pathogens can also cause other maladies [16,17,18]. For instance, EV-A71 is associated with severe central nervous system diseases, such as encephalitis and aseptic meningitis [19,20,21]; while CV-A16 tends to cause more mild HFMD cases. In recent years, CV-A6 is becoming the major causative agent in some areas [22,23,24].

### 1.2. Enteroviruses (EV) Genome and Viral Proteins

The EV genome is a positive-sense single-stranded RNA molecule of 7000–8000 nucleotides composed of a single open reading frame (ORF) with a 5′-untranslated region (5′-UTR) and a 3′-UTR. The 5′-UTR contains an internal ribosomal entry site (IRES) for the binding of the 40S ribosomal subunit to initiate cap-independent translation, while the 3′-UTR contains a pseudoknot and a poly (A) tail. The ORF encodes a large polyprotein precursor, which consists of P1, P2, and P3 regions. In EV infected cells, this polyprotein precursor is initially cleaved between P1 and P2 by viral 2A proteinase, while the P2-P3 junction is cleaved by 3C proteinase [2]. Finally, this precursor is processed into mature viral proteins, including four structural proteins that form the four viral capsid (VP1-VP4) and seven non-structural proteins (2A–2C and 3A–3D). The 2A and 3C proteinase in particular have profound effects on host cells by modulating proteins related with translation, apoptosis, innate immunity, RNA processing and polyadenylation [25,26,27,28,29]. In this review, we will mainly focus on the function of 2A and 3C proteinase to modulate innate immune responses.

## 2. Innate Immunity

### 2.1. Innate Immunity Signaling Pathways

Innate immunity is the first line of host defense against invading pathogens, where type I interferon (IFN) production is initiated at the early stages and subsequently induces the expression of IFN-stimulated genes (ISGs) in an autocrine and paracrine manner, leading to the destruction of invading pathogens [30,31]. Upon infection, type-I IFN is activated by pathogen-associated molecular patterns (PAMPs) which are produced in response to microbial replication, including virus nucleic acid in the form of single-stranded (ss) and double-stranded (ds) DNA, and ss- and dsRNA, viral glycoproteins, bacterial components, fungal cell walls, and flagella proteins [32,33,34]. These PAMPs are recognized by pathogen recognition receptors (PRRs). RNA viruses such as EVs are primarily detected by three types of PRRs: Toll-like receptors (TLRs), retinoic acid-inducible gene (RIG-I)-like receptors (RLRs) and nucleotide-binding oligomerization domain (NOD)-like receptors (NLRs) [33,34].

The TLRs primarily detect PAMPs on the cell surface or the lumen of intracellular vesicles such as endosomes or lysosomes. Endosome-associated TLR3, 7/8 and 9 can recognize viral nucleic acids [33,35]. For instance, TLR3 recognizes viral dsRNA, TLR7/8 recognizes ssRNA, and TLR9 recognizes unmethylated 2’-deoxyribo (cytidine-phosphate-guanosine) (CpG) DNA [35]. However, TLR4 in the plasma membrane was activated by lipopolysaccharide (LPS) [36]. After recognition activity, these TLRs recruit TIR-containing adaptors, such as myeloid differentiation primary response gene 88 (MyD88) and Toll/interleukin-1 receptor domain-containing adaptors inducing IFN-β (TRIF) to transmit signals downstream [37]. In response to viral dsRNA, the TLR3 signaling pathway is activated by the adaptor TRIF, which in turn recruits tumor necrosis factor receptor-associated factor 3 (TRAF3). TRAF3 activates the two related kinases, TANK-binding kinase 1 (TBK1) and inhibitor of κB kinase (IKKi), which mediates phosphorylation of interferon regulatory factor 3/7 (IRF3/7) [38]. Subsequently, IRF3/7 enters the nucleus to stimulate the production of type I IFNs. TLR3 signaling also mediates NF-κB activation by a TRIF-dependent manner. The C-terminal of TRIF interacts with receptor-interacting protein 1 (RIP1). Alternatively, TRIF can also activate NF-κB by directly interacting with TRAF6. TRAF6 further activates the TAK1 complex, which mediates NF-κB and MAP kinase activation [39]. Additionally, TLR7 and TLR8 signals rely on the adaptors MyD88 and TRAF6. The interleukin-1 receptor-associated kinases (IRAK1-4) are then activated to induce phosphorylation of the IRF3/7 and activation of NF-κB [40].

In contrast to the TLRs, two cytoplasmic sensors, including RIG-I and melanoma differentiation-associated gene 5 (MDA5), recognize viral RNA in the cytosol [32]. RIG-I recognizes short dsRNA and ssRNA with a 5-triphosphate; while MDA5 recognizes long dsRNA [41]. Recently, it has been reported that RIG-I cannot recognize endogenous RNA because these self RNAs have a N1-2’*O*-methylation modification, and the yellow fever virus can evade RIG-I recognition by forming 2’*O*-methylation RNA [42]. RIG-I and MDA5 consist of two caspase activation recruitment domains (CARDs), a helicase domain, and a C-terminal domain. The two CARD domains are important for activating type I IFN by interacting with the adaptor mitochondrial antiviral signaling protein (MAVS, also termed as IPS-1, VISA, Cardif). MAVS serves as a scaffold to interact with TRAF3 and TBK1/IKKi complex which activates IRF3/7, leading to activation of type I IFN production [33,34].

Finally, NLRs are also intracellular PRRs that initiate innate immune responses to microbial infection [43]. Certain NLRs can be assembled into larger structures termed inflammasomes, of which the NOD-like receptor, pyrin domain containing 3 (NLRP3) inflammasome is the most studied and well characterized [44,45]. NLRP3 is not constitutively expressed within cells, but is activated by TLR signaling when cells are infected by microbial species. NLRP3 then interacts with the apoptosis-associated speck-like protein containing a CARD adaptor protein (ASC) by the Pyrin domain. ASC in turn recruits pro-caspase-1 and induces auto-cleavage of caspase-1. Activated caspase-1 is crucial for pro-IL-1β and pro-IL-18 processing, and induces secretion of IL-1β and IL-18, which can induce inflammatory responses [46,47].

### 2.2. Innate Detection of Enterovirus

Upon EV infection, viral components are recognized by the three PRRs mentioned above. For instance, it has been reported that TLR7 and TLR8 recognize ssRNA upon CV-B3 infection. Subsequently, MyD88 is recruited into endosomes to further activate the innate immunity downstream response [48]. Thus, TLR7 and TLR8 play critical roles on recognizing CV-B3 ssRNA and CV-B3-induced IFN production, although the precise molecular mechanisms of them remain elusive [48,49,50]. TLR3 signaling in macrophages is responsible for CV-B4-mediated innate immune responses [51]. In TLR3-deficient mice, CV-B4-induced pro-inflammatory mediators are reduced and viral replication is increased, which together leads to severe cardiac damage. These findings suggest that TLR3 signaling on macrophages is responsible for the host immune responses against CV-B4 infection [51]. TLR3 is also important for restricting CV-B3 infections as TLR3^−/−^ mice infected with CV-B3 were found to have increased myocarditis and mortality [52,53]. The role of TLR3 as well as MDA5 in PV infection was tested in TLR3- or MDA5-deficient mice [54]. Although PV-induced IFN production is dependent on MDA5 in cultured primary kidney cells, mice mortality rates were similar between MDA5-deficient- and wild-type mice. However, mortality rates in TLR3-deficient mice were significantly increased compared to wide-type mice upon PV infection [54]. These results indicated that TLR3 is a pivotal component of the innate stress response against EV infection.

TLR9 is also important for immune responses against EV-A71 infection as TLR9 knockout mice display more serious symptoms than wild type mice after EV-A71 infection [55]. Interestingly, EV-A71 infection cannot directly activate TLR9-mediated innate immune responses, which are stimulated by EV-A71-induced danger-associated molecular patterns (DAMPs) release. In addition to TLRs, studies have shown that MDA5 is an important cytoplasmic sensor against CV-B3, EV-A71, and PV infection [56,57,58]. The dsRNA from these viruses-infected cells could activate MDA5 and induce IFNβ production, indicating that MAD5 is important for recognizing EVs [59]. Overexpression of MDA5 and RIG-I also enhance EV-A71 RNA-induced production of type I IFN [57]. These results suggest that MDA5 and RIG-I are involved in the recognition of EV-A71, though additional studies using knockout mice are needed to further elucidate the role of MDA5 and RIG-I under physiological conditions. Similar with these viruses, HRVs are coordinately recognized by constitutively expressed TLR3/TRIF and later inducible RLRs in primary bronchial epithelial cells [60].

Little is known about the role of NLRs in the detection of EV infection. Recently, it has been reported that CV-B3-induced inflammasome activation is NLRP3-dependent. NLRP3 inflammasome plays a critical role on the pathogenesis of CV-B3 [61]. Consistent with this, our results also show that the NLRP3 inflammasome plays a protective role against EV-A71 infection *in vivo* [62].

In summary, TLRs and RLRs signaling are involved in detecting EV infection. However, different species of EVs stimulate host innate immune responses by activating different molecules in PRRs signaling pathways.

## 3. Evasion of Innate Immunity by EVs

**Figure 1:**
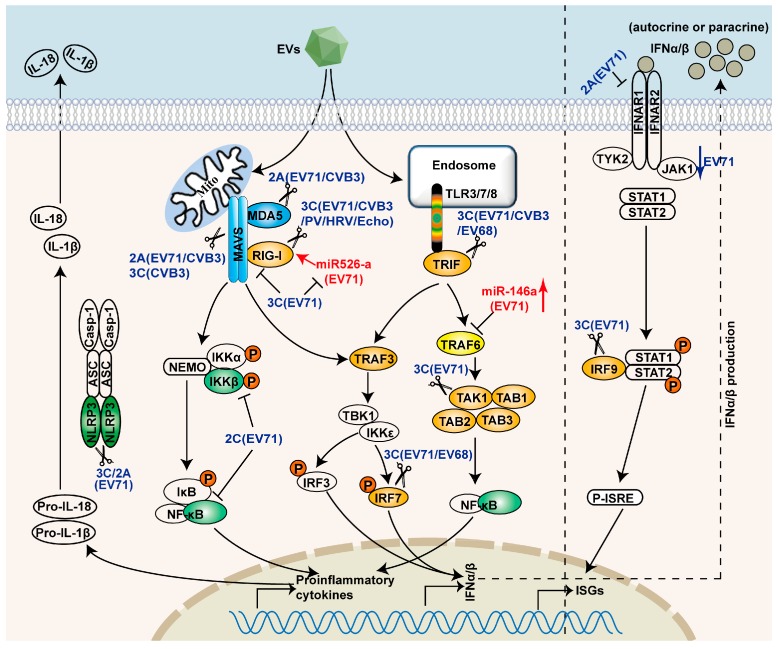
Evasion of PRRs-mediated signaling pathways by EVs. EVs target PRRs signaling pathways by non-structural proteins, 2A, 2C, and 3C. 2A cleaves MAVS and MDA5, and degrades IFNAR1. 2C inhibits NF-κB activation. 3C disrupts the RIG-I and MAVS complex, and cleaves TRIF, IRF9, IRF7, TAK1, and TAB1/2/3. The scissors represent cleavage. 3C also inhibits miR-526a-mediated RIG-I activation. EV71 can also evade innate immunity by inducing the expression of miR-146a.

The innate immune response is critical in controlling EV infection. In order to replicate and survive, many species of EVs have evolved diverse strategies to evade IFN responses. These strategies are discussed in the following sections and summarized in Figure 1 and Table 2.

### 3.1. Shutoff of Host Protein Synthesis

PV infection results in the shutoff of host RNA and protein synthesis. In this process, the eukaryotic translation initiation factor 4G (eIF4G) and the p220 subunit of the cap-binding protein complex are cleaved by PV 2A proteinase [25,63]. These results suggest that PV 2A is related to diminished IFN production. However, eIF4G cleavage only partially induces translation shutoff, indicating additional mechanisms are involved in EV-induced inhibition of translation [64,65,66]. Similarly, EV3C-mediated cleavage of poly(A)-binding protein (PABP) plays important role in translation arrest [67]. PV infection also can inhibit host cell RNA polymerase II-mediated transcription by 3C proteinase mediated cleavage of TATA-binding protein (TBP) and cyclic AMP-responsive element-containing protein (CREB) [68,69]. These findings show that EVs inhibit the IFN signaling pathways, at least in part, by the shutoff of host mRNA transcription and translation through 2A and 3C proteinase.

**Table 2:** Targets of EVs on evading antiviral responses.

Targets	Viral Proteins	Antagonizing Approach	References
RIG-I	EV-A71 3C	Impeding ofinteraction between RIG-I and MAVS	[26]
	CV-B3 3C	Cleavage	[56]
	PV 3C	Cleavage	[56,70]
	Rhinovirus 16 3C	Cleavage	[70]
	Echovirus 3C	Cleavage	[70]
MDA-5	EV-A71 induced caspases	Cleavage	[57]
	PV induced caspases	Degradation in proteasome- and caspase-dependent manner	[71]
	EV-A71 2A	Cleavage	[56]
	CV-B3 2A	Cleavage	[56]
	PV 2A	Cleavage	[56]
MAVS	EV-A71 2A	Cleavage	[28,56]
	PV2A	Cleavage	[56]
	PV induced caspases	Cleavage	[72]
	CV-B3 3C	Cleavage	[73]
	CV-B3 2A	Cleavage	[56]
	HRV1a 2A, 3C and caspases	Cleavage	[74]
TRIF	EV-A71 3C	Cleavage	[75]
	EV-D68 3C	Cleavage	[76]
	CV-B3 3C	Cleavage	[73]
IRF7	EV-A71 3C	Cleavage	[77]
	EV-D68 3C	Cleavage	[78]
IRF9	EV-A71 3C	Cleavage	[79]
IKKβ	EV-A71 2C	Inhibition of phosphorylation	[80]
P65	EV-A71 2C	Inhibition of interaction between P65 and P50	[81]
TAK1 Complex	EV-A71 3C	Cleavage of TAK1/TAB1/TAB2/TAB3	[82]
IFANR1	EV-A71 2A	Down-regulation	[83]
G3BP1	CV-B3 3C	Cleavage	[84]
miR-146a	EV-A71	Upregulation of miR-146a to inhibit IRAK1- and TRAF6-mediated IFNβ production	[85]
miR-526a	EV-A71 3C	Downregulation	[86]

### 3.2. Interference with PRRs Recognition

Given that EVs are detected by pathogen recognition receptors on cell surfaces as well as in the cytosol, EVs have developed diverse strategies to antagonize these receptors. For instance, PV and HRV 1α infection induces the degradation of MDA5 in a proteasome- and caspase-dependent manner. However, this receptor degradation is independent of 2A and 3C protease that seem to be responsible for the inhibition of IFN production against EV infection [71]. Conversely, CV-B3, EV-A71 and PV 2A proteinases directly cleave MDA5, although this seems to be inconsistent with an earlier report that EV-A71 induces MDA5 cleavage in a caspase-dependent manner [56,57,58]. Besides MDA5, RIG-I is another critical target for CV-B3 and EV-A71. Their infections can induce RIG-I cleavage by 3C proteinase [56,70]. These findings indicate that MAD5 and RIG-I are common targets for EVs-mediated evasion against innate immune responses. However, our results show that 3C inhibits RIG-I-mediated type I IFN production by impeding the formation of a functional complex between the cytosolic RIG-I and the adaptor molecule MAVS rather than by cleaving RIG-I directly [26]. After EV-A71 infection, NLRP3 inflammasome is activated and plays a protective role against EV-A71 infection [62]. In turn, EV-A71 infection inhibits NLRP3 inflammasome activation by directly cleaving NLRP3 with 2A and 3C proteinases [62]. Despite the critical role of TLRs on antiviral responses against EVs, to our knowledge there is no evidence that EVs can directly target TLRs to evade innate immunity.

### 3.3. Interference with Adaptor Molecules and Downstream Effectors in Innate Immune Signaling Pathways

As show in Figure 1, many molecules in innate immune signaling pathways are targets for EVs to evade antiviral responses. In addition to interfering with the PRRs, EVs have also developed protective strategies against the downstream adaptor molecule MAVS [28,56,58,72]. For example, EV-A71 2A proteinase directly targets and cleaves MAVS at three distinct sites. None of these cleavage fragments can activate the type I IFN production [28]. In addition, CV-B3 infection can cleave MAVS in a pattern similar to that of EV-A71 infection [28,56]. However, the cleavage fragments of MAVS induced by PV 2A proteinase are different from CV-B3 and EV-A71 [56], indicating that PV 2A may cleave MAVS at different sites. CV-B3 3C proteinase cleaves overexpressed MAVS *in vitro*, while EV-A71 3C does not cleave MAVS [26,28,73]. Furthermore, HRV 2A, 3C and HRV-induced caspases are all closely related to HRV-induced MAVS cleavage [74]. These data suggest that cleavage of MAVS may be a common phenomenon among EVs to antagonize type I IFN induction.

The TLR3-TRIF-mediated signaling pathway is important for antiviral responses against PV infectionin mice [54]. Consistent with this observation, TLR3 signaling activation in macrophages is important for protecting older mice against EV-A71 and CV-A16 infection [87,88]. Accordingly, to survive, EV-A71 inhibits TLR3-mediated antiviral responses by 3C proteinase, which interacts with TRIF and induces its cleavage [75]. In addition, the 3C proteinase of CV-B3 can also cleave TRIF at multiple sites in the N- and C-terminal regions and localized with TRIF to the signalosome complex within the cytoplasm to impede type I IFN production [73]. Furthermore, our recent results show that 3C of EV-D68 can also cleave TRIF although at different sites compared to those from EV-A71 and CV-B3 [76]. These data indicate that TRIF is a common target for EVs to antagonize the TLR3 signaling pathway.

Once adaptor molecules are activated, signals are transmitted to downstream effector targets, including the IκB complex and TBK1/IKKε complex, which activate NF-κB and IRF3/7, respectively. Upon activation, NF-κB and IRF3/7 translocate to the nucleus and induce type I IFN production [33,34]. EV-A71 and EV68 infection decreases the expression of IRF7 [77,78]. The EV-A71 3C proteinase directly induces IRF7 cleavage at Q189–S190, which is dependent on 3C proteolytic activity. The two cleavage fragments are incapable of activating type I IFN production [77]. Consistent with these studies, Lee *et al.* [89] show that the EV-A71 3C proteinase inhibits type I IFN synthesis in mice.

Viruses can also inhibit innate immune responses by targeting the NF-κB signaling pathway, which is important in activating the production of IFNs or inflammatory cytokines. Our results show that EV-A71 3C proteinase inhibits NF-κB activation by cleavage of the transforming growth factor-β-activated kinase 1 (TAK1) complex [82]. In addition to the 2A and 3C proteinases, the non-structural protein 2C is also important for EV-A71to evade innate immunity [80,81]. Specifically, this 2C protein interacts with IKKβ, which inhibits the activation of NF-κB mediated by TNF-α [80]. Recently, it has been reported that the 2C protein of EV-A71, EV-D68, and PV also inhibits NF-κB activation by interacting with p65. This interaction inhibits the formation of heterodimer p65 and p50, the most abundant member of NF-κB family [81]. In summary, these data indicate that EVs also evade innate immunity by way of the 2C protein.

Taken together, the non-structural proteins 2A and 3C proteinases are important factors by which EVs inhibit IFN and thus evade innate immune responses (Table 2). Additionally, 2C also plays critical roles involved in innate immunity.

### 3.4. Interference with IFN-Mediated Signaling

The studies mentioned above illustrate that EV-A71 inhibits the production of IFNs at various points. Does EV-A71 also affect activation of the IFN-1 receptor (IFNAR1)? The activity of this receptor induces interferon-stimulated genes (ISGs) via the Janus activated kinase (Jak)-signal transducers and activators of transcription (STAT) signaling pathways. Indeed, Lu *et al.* [83] found that the 2A proteinase of EV-A71 can degrade IFNAR1, which in turn inhibits the IFN-mediated phosphorylation of STAT1, STAT2, Jak1, and tyrosine kinase 2 (Tyk2). However, Liu *et al.* [90] showed that EV-A71 inhibits JAK1-STAT signaling by down-regulating JAK1 rather than IFNAR1. IRF9, a component of the heterocomplex IFN-stimulated gene factor 3 (ISGF3) that induces the expression of ISGs, is another target of EV-A71 for blocking JAK1-STAT signaling, which is cleaved by EV-A71 3C proteinase [79].

### 3.5. Interference of Other Signaling Pathways

EVs can also evade innate immunity by interfering with other signaling pathways. For instance, early stages of PV and CV-B3 infections induce the formation of stress granules, which have antiviral activity and mediate innate immune signaling through the Ras-GTPase-activating protein (SH3 domain) binding protein 1 (G3BP1) [91,92,93,94]. However, in later stages of infection the stress granules disappear after the 3C proteinase cleaves G3BP1, a stress granule-nucleating protein [84,93]. These findings indicate that EVs may hinder the formation of stress granules or disassemble them to counter the antiviral activity of stress-granules through cleaving G3BP1.

EVs can also mediate innate immune responses by regulating miRNA functions [85,86], which have critical roles in regulating EV-host interactions [95,96,97]. For instance, EV-A71 infection can suppress IFN production by inducing the expression of miR-146a [85]. The miR-146a is a negative-feedback regulator of RLR signaling, which can inhibit the expression of IRAK1 and TRAF6, two major elements in IFN production [85]. Recently, Xu *et al.* [86] showed that RIG-I signaling pathway can be positively regulated by EV-A71-induced miR-526a. However, in later stages of EV-A71 infection, the 3C proteinase can block the expression of miR-526a and further inhibit IFN production. Other miRNAs are also involved in EV infections, including EV-A71, CV-B3, and HRV [97]. However, to date, there are few miRNAs as targets for EVs to escape innate immune responses.

## 4. Roles of Innate Immunity Evasion in EV Pathogenesis

Innate immunity is important for the control of the EV infection at the early stage, as evidence show that EV-induced morbidity and mortalityis increased in type I IFNs- or IFNAR-knockout mice [98,99]. In addition, treatment with neutralizing antibody of type I IFN increases viral loads and EV-A71-induced lethality while type I IFN treatment increases the survival rate of mice [99]. These findings indicate that the innate immune responses are closely associated with EV pathogenesis. However, there is little direct evidence demonstrating how these mechanisms to antagonize and evade innate immunityare related to EV pathogenesis. To gain further insights into these mechanisms, it will be useful to examine whether recombinant EV that contains mutated 2A, 3C, or 2C, which are unable to antagonize the described targets, can induce stronger IFN response compared with wild type virus. Furthermore, understanding the molecular mechanisms by which EVs hijack the PRRs signaling pathways would offer clues for anti-EVs drug design.

## 5. Conclusions

As innate immunity is the first line of defense against viral infections, viruses must develop mechanisms to evade innate immune responses to survive. The interaction between EVs and host is extremely complicated. As discussed in this review, host recognizes the viruses by three types of PRRs and initiates type I IFN response upon EV infection. At the same time, EVs employ a variety of strategies to evade the host innate immune response to ensure an effective infection, which can lead to viral pathogenesis. However, the immune-evasion of EVs is still not fully understood. Which target(s) is/are essential for effective inhibition? At which stage do these non-structural proteins target these molecules under physiological conditions? Furthermore, many of the reports on cell-virus interaction is detected in cell lines which grow very fast and allow high level virus replication, while in certain primary cells the amount of progeny virus is reduced compared to cell lines, this could be the reason for differences in the interactions between the viral proteins and the host proteins. Hence the innate immunity response and viral evasion in primary cells should be investigated. Future studies directed at these questions and assessing the role of particular host proteins will assist in our understanding of EV pathogenesis.
